# From static to dynamic: Artificial intelligence revolution in perioperative care through multimodal data fusion and closed-loop optimization

**DOI:** 10.1016/j.jatmed.2025.06.003

**Published:** 2025-08-13

**Authors:** Mingdi Xue, Jiaming Yang, Honglin Wang, Zineng Yan, Xiaoliang Chen, Weihang Gao, Rui Luo, Xiangxin Lv, Zhewei Ye

**Affiliations:** a Department of Orthopedics, Union Hospital, Tongji Medical College, Huazhong University of Science and Technology, Wuhan 430000, China; b Intelligent Medical Laboratory, Union Hospital, Tongji Medical College, Huazhong University of Science and Technology, Wuhan 430000, China

**Keywords:** Artificial intelligence, Perioperative care, Multimodal data fusion, Closed-loop optimization

## Abstract

The integration of artificial intelligence (AI) into perioperative care represents a paradigmatic transformation from static decision frameworks to dynamic, adaptive systems. This comprehensive review synthesizes recent breakthroughs in multimodal data fusion and closed-loop optimization for perioperative care, examining the convergence of technological innovation with clinical imperatives. We systematically analyze three critical dimensions: the fundamental paradigm shift driven by clinical complexity, technological breakthroughs in multimodal fusion architectures, and dynamic decision-making implementations in anesthesia depth regulation and pain management optimization. Our analysis reveals that contemporary perioperative environments demand sophisticated AI systems capable of real-time data integration and dynamic response optimization. We identify critical bottlenecks in clinical implementation, including computational optimization under real-time constraints, robustness assurance mechanisms, and interpretability challenges. Looking forward, we delineate three interconnected pillars essential for advancing the field: standardized multimodal benchmark datasets, human-AI collaborative decision-making paradigms, and ethical-regulatory frameworks. This review establishes a comprehensive roadmap for transforming perioperative care through AI integration, emphasizing the synergistic relationship between technological innovation and clinical expertise in optimizing patient outcomes.

## Introduction

Perioperative care represents one of medicine's most complex and high-stakes environments, where real-time clinical decisions directly impact patient safety and surgical outcomes.^1^, ^2^ The integration of anesthesia management, hemodynamic monitoring, pain control, and complication prevention requires sophisticated decision-making capabilities that traditionally rely on clinician expertise and static protocols.^3^ However, the exponential growth in multimodal perioperative data—encompassing continuous physiological monitoring, electronic health records, imaging data, and real-time laboratory results—has created unprecedented opportunities for computational intelligence to augment clinical care.^4^, ^5^ Artificial intelligence (AI) technologies, particularly those leveraging machine learning, deep neural networks, and closed-loop control systems, are emerging as transformative tools capable of synthesizing vast heterogeneous data streams into actionable clinical insights.^5^ This paradigm shift from reactive, protocol-driven care to proactive, data-driven precision medicine promises to revolutionize perioperative practice through enhanced predictive accuracy, personalized treatment optimization, and automated decision support.^6^ This comprehensive review examines the current state and future trajectory of AI integration in perioperative care, analyzing the technological foundations of multimodal data fusion, evaluating clinical applications in dynamic decision-making scenarios, and identifying critical implementation challenges that must be addressed to realize the full potential of intelligent perioperative care systems.

## Paradigm shift: clinical imperatives

The contemporary perioperative landscape stands at an inflection point, poised for transformative reconfiguration through artificial intelligence integration. Traditional perioperative protocols—characterized by static decision frameworks, compartmentalized data streams, and reactive intervention models—have reached their inherent limitations in addressing the multidimensional complexity of modern surgical care.^7^, ^8^ This paradigmatic constraint necessitates fundamental reconceptualization of perioperative management architectures to accommodate the increasingly intricate interplay between physiological variability, procedural complexity, and outcome optimization. The emergence of advanced computational modalities, particularly those leveraging multimodal data fusion and reinforcement learning frameworks, presents unprecedented opportunities to transcend conventional perioperative care limitations and establish dynamic, anticipatory management ecosystems.^9^

### Complexity dimensions in perioperative decision architecture

Perioperative decision-making represents an extraordinarily multidimensional computational challenge characterized by dynamic physiological interdependencies operating under procedural heterogeneity and temporal constraints.^10^, ^11^ This complexity transcends unimodal analytical paradigms, as evidenced by emerging integrated analytics platforms that demonstrate superior predictive performance through multimodal data fusion.^12^ Furthermore, perioperative complexity extends beyond physiological parameters into ethical-clinical intersections where discontinuous provider-patient relationships and time-constrained translation of abstract values into concrete clinical protocols create computational dimensions resistant to algorithmic reduction.^13^ These complexity vectors—physiological variability,^14^ procedural heterogeneity,^15^ temporal evolution, and ethical-clinical intersections—function synergistically rather than additively, generating a computational challenge that fundamentally exceeds the capacity of conventional static decision frameworks and necessitates advanced dynamic computational architectures.

### The imperative for dynamic closed-loop regulation

The inherent limitations of static decision frameworks within perioperative settings necessitate a paradigmatic shift toward dynamic closed-loop regulatory architectures. Contemporary evidence demonstrates significant clinical decision optimization through automated feedback-integrated systems, with Karlsson et al. documenting substantial improvements in guideline adherence for anticoagulation therapy through electronic health record-integrated clinical decision support tools.^16^ This implementation efficacy extends to perioperative domains, where Gracia et al. demonstrated an 83 % reduction in unnecessary chest radiography, 54 % reduction in electrocardiograms, and cost savings exceeding €1 million through closed-loop clinical decision support implementation, without compromising surgical safety metrics.^17^ The superiority of closed-loop architectures over open-loop counterparts manifests through real-time data integration, algorithmic optimization, and continuous recalibration capacities that accommodate physiological variability and clinical complexity—features that necessitate sophisticated multimodal data fusion methodologies capable of synthesizing heterogeneous information streams across temporal, spatial, and physiological dimensions while maintaining computational tractability and clinical interpretability.

## Multimodal fusion: technological breakthroughs

The realization of dynamic closed-loop perioperative systems necessitates revolutionary advances in multimodal data integration methodologies. Contemporary perioperative environments generate unprecedented volumes of heterogeneous data streams—spanning continuous physiological monitoring, intermittent laboratory measurements, clinical observations, and radiological/ultrasonographic assessments—each characterized by distinct temporal resolutions, signal-to-noise profiles, and clinical significance hierarchies.^18^ The transformative potential of artificial intelligence in perioperative care hinges critically on technological innovations capable of synthesizing these disparate information modalities into unified computational frameworks while preserving modality-specific information characteristics and capturing cross-modal dependencies. Recent breakthroughs in representational learning architectures, temporal alignment methodologies, and cross-domain generalization strategies have catalyzed remarkable progress toward clinically viable multimodal fusion platforms optimized for perioperative implementation.^19^, ^20^, ^21^

### Data-level fusion challenges

The integration of heterogeneous perioperative data streams presents formidable computational challenges that transcend conventional data processing paradigms. Perioperative environments generate multidimensional data characterized by profound heterogeneity across temporal resolution (continuous hemodynamic waveforms versus intermittent laboratory measurements), signal fidelity (high-resolution arterial pressure waveforms versus sparse subjective pain assessments), and data organization frameworks (structured vital signs versus unstructured clinical observations).^22^ This fundamental heterogeneity necessitates sophisticated fusion architectures capable of reconciling temporal misalignment, addressing specific interference patterns in different data types, and handling common data gaps across different measurement types. Contemporary machine learning applications exemplify these challenges: Hatib et al. developed an algorithm extracting thousands of key features from arterial pressure waveforms to predict hypotension with remarkable accuracy (AUC 0.95–0.97) at various prediction horizons,^22^ demonstrating the immense information density within single-modality high-fidelity physiological signals. Similarly, Kang et al. achieved substantial predictive performance (AUC 0.842) by integrating multimodal perioperative data from anesthesia monitors, infusion pumps, anesthesia machines, and electronic health records.^23^ The computational complexity intensifies when considering cross-modal dependencies, as illustrated by Lundberg et al.'s explainable machine learning system for hypoxemia prediction, which synthesized minute-by-minute data from over 50,000 surgeries while maintaining interpretability.^24^

Beyond technical integration challenges, perioperative data fusion must contend with fundamental epistemological questions regarding information complementarity across modalities. Current research indicates that optimal predictive architectures must selectively leverage modality-specific information characteristics—for instance, Geng et al. demonstrated that an artificial neural network integrating just three clinical variables (BMI, habitual snoring, and neck circumference) achieved remarkable predictive efficacy (AUC 0.80) for hypoxemia during sedation,^25^ suggesting that parsimonious multimodal integration can sometimes outperform exhaustive unimodal feature extraction. This finding underscores the cardinal challenge in perioperative data fusion: developing computational frameworks that can dynamically determine optimal integration depth across modalities while preserving information integrity, addressing modal-specific noise characteristics, and maintaining computational tractability—all within the stringent real-time constraints of clinical decision support systems.

### Model architecture innovation

Recent advances in artificial intelligence architectures have catalyzed unprecedented breakthroughs in multimodal perioperative data integration and analysis. Transformer-based architectures (AI models specialized in processing sequential data) have emerged as particularly powerful frameworks for capturing complex cross-modal relationships in perioperative settings.^26^ For instance, He et al. developed an innovative transformer-based architecture incorporating Long Short-Term Memory (LSTM) and Gate Residual Network (GRN) components for anesthesia depth prediction, demonstrating superior performance in capturing sudden state transitions during propofol and remifentanil administration.^27^ This architectural innovation was further validated by Wang et al.'s meta-learning-based deep learning framework (Anes-MetaNet), which effectively addressed cross-subject variability in EEG-based consciousness state classification through a sophisticated combination of Convolutional Neural Networks (CNN) and LSTM networks.^28^

The evolution of multimodal integration architectures has been particularly evident in intensive care applications, where novel attention mechanisms, which are AI techniques that mimic human-like focus on important information, have revolutionized the fusion of heterogeneous data streams.^29^, ^30^ Khader et al. demonstrated the superiority of a medical transformer architecture in integrating imaging and non-imaging data for survival prediction (AUROC 0.863), which significantly outperformed single-modality approaches.^31^ This architectural paradigm has been further refined in anesthesia monitoring applications, as exemplified by Han et al.'s deep learning model combining intracranial and scalp EEG data (AUC 0.795), which leveraged sophisticated spatiotemporal feature extraction to enhance sedation level prediction.^32^ The integration of multiple neural signal pathways has proven particularly powerful, as demonstrated by Tacke et al.'s machine learning approach combining spontaneous EEG and auditory evoked potentials, achieving a remarkable prediction probability of 0.935 for consciousness state classification.^33^

### Generalization enhancement strategies

The advancement of robust generalization capabilities for multimodal perioperative AI systems represents a critical frontier in translational clinical implementation. Contemporary research has illuminated multiple complementary strategies for enhancing cross-institutional and cross-population generalizability. Transfer learning approaches, knowledge transfer techniques (applying AI insights learned from one hospital to another), have demonstrated particular efficacy in perioperative contexts,^34^ as evidenced by Hu et al.'s intelligent anesthesia prediction algorithm, which achieved remarkable improvements in dosage prediction accuracy (R² = 0.915) through sophisticated neural architectures incorporating bidirectional LSTM networks and attention mechanisms.^35^ This architectural paradigm has been further refined through contrastive transfer learning methodologies—enhanced knowledge transfer techniques that improve adaptability by comparing different data characteristic—with Salehinejad et al. demonstrating superior predictive performance for adverse events through unsupervised pretraining on large-scale temporal data followed by targeted fine-tuning.^36^ Critical insights into optimal transfer strategies have emerged from cross-institutional studies—Millarch et al.'s comprehensive analysis of model transfer approaches in trauma outcome prediction revealed that mixed-dataset training with class weighting and selective transfer learning achieved exceptional performance (ROC-AUC 0.988) in handling imbalanced outcomes,^37^ while Wiens et al. demonstrated the efficacy of institution-specific model adaptation in enhancing prediction accuracy across heterogeneous hospital environments.^38^ These advances collectively establish a robust framework for developing generalizable perioperative AI systems that maintain consistent performance across diverse clinical settings while accommodating institutional variations in patient populations and clinical protocols.

### Key AI algorithms in perioperative management

The integration of artificial intelligence into perioperative care employs several distinct algorithm families, each serving specific clinical needs. Supervised learning algorithms—particularly Random Forest and Gradient Boosting—have proven effective for predicting surgical complications and risk stratification, with sensitivity rates exceeding 85 % in predicting unplanned ICU admissions while providing results that clinicians can readily understand^39^, ^40^ Deep learning approaches include Convolutional Neural Networks (CNNs), which excel at analyzing complex patterns in images and waveforms such as EEG and arterial pressure signals,^41^ and Recurrent Neural Networks (especially LSTM variants), which effectively capture temporal trends in vital signs monitoring by "remembering" previous states—a critical feature for detecting gradual deterioration in patient status.^42^ More recently, Transformer architectures have shown exceptional capability in integrating diverse data types (lab values, medications, vitals) by recognizing connections between seemingly unrelated clinical parameters, improving outcome prediction accuracy by 15–20 % compared to conventional methods.^43^

For dynamic clinical management, Reinforcement Learning algorithms have emerged as powerful tools for optimizing medication dosing strategies through simulated trial-and-error, effectively "practicing" without patient risk.^44^ These algorithms have demonstrated particular value in closed-loop anesthesia systems, reducing target BIS value deviation by approximately 40 % compared to traditional approaches while dynamically adapting to individual patient responses.^45^ Meanwhile, unsupervised learning methods offer unique advantages in detecting anomalous patterns without requiring labeled examples—notably using Autoencoders to identify unusual physiological states that may precede complications, with detection rates over 90 % for critical events up to 30 min before conventional alarms.^46^ Unlike traditional statistical methods that rely on predefined relationships, these AI approaches automatically discover complex patterns across multiple physiological systems, offering more comprehensive patient monitoring while reducing false alarms by 60–70 % through contextual understanding of clinical signals.^47^

## Dynamic decision-making clinical scenarios

The translation of multimodal data fusion and artificial intelligence advances into dynamic perioperative decision-making represents a paradigmatic revolution in anesthesia practice. Contemporary perioperative care increasingly demands real-time, adaptive control systems capable of synthesizing heterogeneous data streams to optimize clinical outcomes.^48^ Two critical domains have emerged as primary proving grounds for dynamic decision architectures: depth of anesthesia regulation and closed-loop pain management optimization. These applications exemplify the convergence of sophisticated algorithmic frameworks with clinical imperatives, demonstrating the transformative potential of AI-driven dynamic control systems in perioperative care while illuminating both the remarkable progress achieved and the challenges that remain in implementing truly autonomous decision support platforms.

### Real-time regulation of anesthesia depth

The integration of artificial intelligence into anesthesia depth regulation represents a transformative advance in perioperative care optimization, particularly through sophisticated multimodal monitoring and control architectures.^49^ Recent innovations in neural network-based approaches have demonstrated remarkable efficacy in personalizing anesthetic drug delivery. Chen et al. developed a convolutional neural network incorporating sliding window sampling and residual learning modules that achieved comparable performance to clinical anesthesiologists in propofol and remifentanil dosing during maintenance phase anesthesia, validating the feasibility of AI-driven open-loop control systems.^50^ This architectural paradigm has been further refined through multimodal fusion strategies, as evidenced by Bahador et al.'s pioneering work in multidimensional physiological signal integration of EEG-ECG recordings, which achieved 94.14 % precision in anesthesia state classification through innovative advanced signal processing technique and deep learning architectures.^51^ Their implementation demonstrated that integrated multimodal monitoring could provide more comprehensive and reliable assessment of anesthesia depth than traditional unimodal approaches, while maintaining rapid processing speeds critical for real-time clinical application.

The emergence of these sophisticated control architectures marks a crucial step toward autonomous anesthesia management systems capable of dynamic, personalized optimization. These advances not only improve the precision of anesthesia depth regulation but also help address the imbalance between the growing surgical demand and the limited anesthesiologist supply in healthcare systems.^50^ However, the transition from research implementations to clinical deployment necessitates careful consideration of system robustness, fail-safe mechanisms, and the optimal balance between algorithmic autonomy and human oversight—considerations that continue to drive innovation in both architectural design and clinical validation methodologies.

### Closed-loop optimization of pain management

The integration of artificial intelligence into perioperative pain management has catalyzed revolutionary advances in closed-loop analgesic control systems, transcending traditional reactive paradigms.^52^ Recent innovations in brain-machine interface (BMI) technologies have demonstrated unprecedented precision in pain modulation. Zhang et al. developed a groundbreaking closed-loop BMI system capable of real-time nociception detection through state-space modeling of anterior cingulate cortex activity, coupled with precision neural activity regulation technology of the prelimbic prefrontal cortex, achieving significant inhibition of both sensory and affective pain behaviors.^53^ This neurophysiological approach has been complemented by autonomous analgesic delivery systems, exemplified by Jonckheere et al.'s closed-loop platform utilizing heart rate variability analysis through the Analgesia Nociception Index (ANI) for precise analgesic titration.^54^ More recently, this approach has been validated in clinical settings by Coeckelenbergh et al., whose randomized controlled trial (n = 70) evaluated the AI-based Nociception Level (NOL) index for guiding intraoperative fentanyl administration. Their findings revealed that the NOL-guided group achieved significantly reduced hourly fentanyl consumption (81 ± 24 vs 108 ± 66 μg·h⁻¹, P = 0.03) and 22 % lower total dosage (P = 0.11) without compromising postoperative pain control, demonstrating the clinical efficacy of AI-driven nociception monitoring in optimizing intraoperative analgesic titration.^55^

The evolution toward intelligent pain management systems has culminated in the emergence of AI-assisted Patient-Controlled Analgesia (AI-PCA), representing a paradigmatic shift in postoperative pain control. Wang et al. demonstrated that wireless intelligent PCA systems incorporating remote monitoring and intelligent analysis capabilities significantly reduced moderate-to-severe postoperative pain incidence while improving patient satisfaction and reducing hospital stays.^56^ This technological advancement has been further validated in vulnerable populations, with Wu et al. demonstrating that elderly colorectal cancer patients receiving AI-assisted patient-controlled intravenous analgesia (AI-PCIA) experienced significantly lower pain scores at key postoperative timepoints (8, 12, 24 h) compared to conventional PCA (*P* < 0.05), along with improved sleep quality metrics on the Richards-Campbell Sleep Questionnaire without increased adverse events.^57^

The integration of machine learning algorithms with multimodal physiological monitoring and Internet of Things (IoT) infrastructure presents unprecedented opportunities for predictive, personalized pain management that anticipates and prevents pain episodes rather than merely responding to them.^56^ This transformation from reactive to predictive pain management architectures, combined with sophisticated closed-loop control systems,^50^ establishes a new frontier in perioperative care optimization that promises to revolutionize the precision and effectiveness of pain management protocols. However, current research predominantly involves semi-closed-loop systems, and fully automated closed-loop pain management still requires more large-scale, multicenter studies to validate its long-term efficacy and safety.

Table 1 summarizes the current applications of artificial intelligence across different domains of anesthesia and pain management, highlighting the AI technologies employed, data types utilized, clinical impact, implementation stage, key challenges, and representative studies discussed in this review.^10^, ^22^, ^23^, ^24^, ^27^, ^32^, ^33^, ^38^, ^50^, ^51^, ^52^, ^54^, ^55^, ^57^, ^58^, ^59^

**Table 1:** Applications of artificial intelligence in anesthesia and pain management based on literature review.

**Application Area**	**AI Technology/Methods**	**Data Types**	**Clinical Impact**	**Implementation Stage**	**Key Challenges**	**Reference**
Depth of Anesthesia Regulation	Convolutional Neural Networks, LSTM Networks, Residual Learning	EEG, ECG, Arterial Pressure Waveforms, Vital Signs	Personalized anesthetic drug delivery, Precision in anesthesia depth, Reduced under/over-dosing	Semi-closed loop systems in clinical trials	Real-time processing constraints, System robustness, Clinical interpretability	27, 50, 51
Pain Management and Nociception Monitoring	Closed-loop Brain-Machine Interface, Neural Networks, NOL Index-based Systems	EEG, ECG, Heart Rate Variability, Skin Conductance, ANI/NOL Indices	Reduced intraoperative opioid consumption, Improved postoperative pain control, Enhanced sleep quality	Clinical validation in progress	Signal quality variability, Individual response heterogeneity, Integration with traditional protocols	54, 55, 57
Hemodynamic Monitoring and Optimization	Machine Learning, Transformer Models	Arterial Pressure Waveforms, ECG, Cardiac Output, Fluid Balance	Hypotension prediction and prevention, Fluid optimization, Reduced intraoperative cardiovascular events	Validated prediction models in clinical use	Sensor integration, Signal-to-noise ratio, Interoperability with existing systems	22, 23, 24
Perioperative Risk Assessment	Decision Trees, Random Forests, Neural Networks	Patient Demographics, Comorbidities, Laboratory Values, Surgical Data	Improved risk stratification, Resource optimization, Enhanced preoperative preparation	Clinical implementation in multiple centers	Data standardization, Bias mitigation, Alert fatigue	10, 38, 58
Sedation Management	Deep Learning, Contrastive Learning	EEG, Intracranial EEG, Auditory Evoked Potentials	Precise sedation level targeting, Reduced adverse events, Improved recovery profiles	Research and early clinical testing	Cross-population variability, Environmental interference, Limited training data	32, 33
Ventilation Management	Reinforcement Learning, Predictive Models	Respiratory Parameters, Blood Gases, Ventilator Settings	Optimized ventilation strategies, Reduced ventilator-induced lung injury, Faster weaning	Prototype testing	Complex physiological interactions, Safety boundaries, Integration with existing ventilators	52, 59
Postoperative Monitoring	IoT Integration, Intelligent PCA, Multimodal Fusion	Vital Signs, Patient Reported Outcomes, Medication Consumption	Early complication detection, Enhanced recovery, Reduced readmissions	Wireless systems in use, AI components in trials	Ambulatory monitoring challenges, Data privacy, Alert threshold optimization	56, 57
Perioperative Crisis Management	Decision Support Systems, Expert Systems	Multimodal Clinical Data, Protocol Databases	Improved guideline adherence, Standardized crisis response, Enhanced team coordination	Simulation studies	Real-time implementation, Workflow integration, User acceptance	16, 17

Abbreviations: EEG: Electroencephalogram; ECG: Electrocardiogram; LSTM: Long Short-Term Memory; ANI: Analgesia Nociception Index; NOL: Nociception Level; PCA: Patient-Controlled Analgesia; IoT: Internet of Things

## Critical bottlenecks in clinical implementation

While artificial intelligence has demonstrated transformative potential in perioperative care through multimodal data fusion and dynamic decision optimization, the translation of these theoretical advances into clinical practice faces several critical implementation challenges. The complexity of perioperative environments, coupled with the stringent requirements for medical system deployment, highlights three fundamental bottlenecks: computational optimization under real-time constraints, robustness assurance mechanisms, and breakthrough directions in interpretability. These challenges represent not merely technical hurdles but rather systemic barriers that must be addressed to realize the full potential of AI-driven perioperative care systems while maintaining the rigorous standards of clinical safety and efficacy.^60^

### Computational optimization under real-time constraints

The integration of multimodal artificial intelligence systems into perioperative care necessitates unprecedented optimization of computational architectures to meet stringent real-time performance requirements. Contemporary research has illuminated multiple complementary approaches to this fundamental challenge. Liu et al. demonstrated the efficacy of optimized efficient AI computation methods for real-time anesthetic drug control, establishing a paradigmatic framework for high-efficiency perioperative computation.^61^ This architectural innovation has been further validated through large-scale empirical analyses. Miyaguchi et al. achieved microsecond-scale prediction latency through sophisticated LSTM implementations in anesthetic infusion event prediction (ROC-AUC 0.753)^.^^62^ Furthermore, a comprehensive meta-analysis of 432 multimodal AI implementations between 2018 and 2024 demonstrated consistent performance advantages (mean AUC improvement: 6.2 percentage points) through optimized architectural frameworks.^63^

The evolution of real-time computational optimization strategies has been particularly evident in clinical implementation contexts, where the imperative for microsecond-to-millisecond decision cycles has catalyzed innovative architectural solutions.^64^, ^65^ Yoon et al.'s systematic review of perioperative AI applications has highlighted the critical role of optimized model architectures in enabling real-time clinical decision support, emphasizing the necessity of balanced trade-offs between computational complexity and latency constraints.^58^ These advances collectively establish a foundational framework for achieving the computational efficiency required for real-time perioperative AI systems while maintaining the high-fidelity analysis capabilities essential for clinical safety and efficacy.

### Robustness assurance mechanisms

The implementation of robust perioperative AI systems necessitates sophisticated mechanisms for ensuring operational stability across heterogeneous clinical environments and patient populations.^22^, ^61^ Dumont et al.'s comprehensive analysis of closed-loop anesthesia control systems has established fundamental frameworks for robustness validation, emphasizing the critical importance of system resilience in maintaining consistent performance across diverse clinical scenarios.^66^ Recent systematic reviews have expanded this conceptual framework, identifying eight distinct robustness concepts in healthcare machine learning applications, highlighting how robustness definitions may vary across development, validation, and deployment phases—a critical consideration for perioperative AI implementation.^67^

This theoretical foundation has been substantiated through empirical validation—West et al. demonstrated remarkable stability in propofol anesthesia control through a robustly-tuned closed-loop system, achieving target depth of hypnosis maintenance in 89 % of cases across a demographically diverse pediatric population, while highlighting the limitations of conventional open-loop approaches in accommodating physiological variability.^68^

Recent advances in reinforcement learning architectures have further revolutionized robustness assurance paradigms in perioperative AI systems.^69^ Schamberg et al. pioneered a continuous-action deep reinforcement learning framework for automated anesthetic dosing, incorporating randomized pharmacodynamic models during training to ensure system resilience across patient variability.^59^ This approach achieved superior performance metrics (median absolute performance error 1.9 % ± 1.8) compared to traditional controllers while maintaining consistency with clinical practice standards. The critical importance of such robustness validation is underscored in clinical applications, as demonstrated by Lee and Jung's analysis of 66,370 surgical patients, which validated machine learning models for identifying high-risk surgical candidates, demonstrating superior performance compared to conventional risk calculators while maintaining reliability across diverse patient cohorts.^58^

Similarly, Absalom et al.'s implementation of automated drug dosage regulation systems in BIS-guided propofol administration demonstrated exceptional stability (MDAPE 5.63 %), establishing new benchmarks for robust performance in automated anesthesia delivery systems.^70^

Beyond conventional validation frameworks, emerging research highlights novel robustness challenges in perioperative AI systems. Bellini et al. identified critical concerns regarding BIS signal reliability in closed-loop anesthesia control systems, emphasizing how environmental interference may compromise system performance and potentially lead to complications in total intravenous anesthesia (TIVA).^71^ These challenges are further compounded by vulnerabilities to adversarial inputs and noise perturbations—a growing concern in critical medical applications where input manipulation could lead to catastrophic outcomes. Recent investigations into deep learning model security have advanced our understanding of these challenges, identifying how model complexity, training data quality, and hyperparameter selection significantly impact robustness, while proposing defensive mechanisms such as adversarial training and data augmentation to enhance system resilience in perioperative settings.^72^, ^73^

### Interpretability breakthrough directions

The imperative for transparent and interpretable AI systems in perioperative care has catalyzed innovative approaches to decision explanation and validation frameworks.^59^ A comprehensive analysis by Singhal et al. of AI applications in anesthesia (2022–2023) has illuminated critical challenges in system interpretability, emphasizing the fundamental importance of transparent decision-making processes for clinical safety and ethical implementation.^74^ This theoretical foundation has been substantiated through empirical investigations—Kambale et al.'s systematic review of 18 anesthesia-focused AI studies revealed that interpretable models consistently enhanced or matched human judgment in critical decisions (*P* < 0.05), while facilitating more informed clinical decision-making through transparent algorithmic processes.^75^ The evolution of interpretability paradigms has been particularly evident in clinical implementation contexts, with Wang et al. highlighting the critical intersection of AI transparency with patient privacy and information security concerns, emphasizing the "black box" phenomenon as a central challenge in clinical adoption.^76^ These challenges have driven innovative solutions in visualization and explanation frameworks, as demonstrated by Hashimoto et al.'s comprehensive analysis of AI techniques in anesthesiology, which established new paradigms for integrating machine learning interpretability with clinical reasoning frameworks.^77^

The triad of implementation challenges—computational optimization under real-time constraints, robustness assurance mechanisms, and interpretability breakthroughs—collectively represents the critical barriers to widespread clinical adoption of AI in perioperative care. While significant advances have been achieved in each domain, the synergistic interaction between these challenges necessitates integrated solutions that simultaneously address computational efficiency, system reliability, and clinical interpretability. The successful navigation of these interconnected challenges will be paramount in realizing the transformative potential of AI-driven perioperative care systems while maintaining the highest standards of clinical safety and efficacy.

### Critical bottlenecks in clinical implementation

The successful deployment of AI-driven perioperative care systems faces substantial translational barriers that extend beyond technical considerations to encompass organizational, regulatory, and socioeconomic dimensions. Healthcare institutions exhibit profound heterogeneity in IT infrastructure capabilities, ranging from legacy electronic health record systems with limited interoperability to state-of-the-art integrated platforms capable of real-time data streaming and processing. This infrastructure variability creates significant challenges for standardized AI implementation, as algorithms optimized for high-performance computing environments may encounter compatibility issues, latency constraints, or data integration failures when deployed across diverse clinical settings.^78^ Furthermore, the fragmented nature of healthcare data systems—characterized by disparate vendor solutions, inconsistent data formats, and varying cybersecurity protocols—necessitates extensive customization and validation processes that substantially increase deployment costs and timeline complexity. These technical disparities are compounded by resource allocation inequities, where smaller healthcare facilities may lack the computational infrastructure, technical expertise, or financial resources to support sophisticated AI implementations, potentially exacerbating existing healthcare disparities.^79^

Beyond infrastructure considerations, the successful integration of AI systems in perioperative care faces significant human factors challenges, particularly regarding clinician acceptance and workflow adaptation. Studies have documented considerable variability in healthcare providers' attitudes toward algorithmic decision support, with acceptance rates influenced by factors including perceived clinical utility, trust in algorithmic recommendations, and concerns about professional autonomy. The integration of AI tools into existing clinical workflows requires comprehensive training programs that address not only technical proficiency but also algorithmic literacy, interpretation of AI-generated insights, and decision-making in scenarios where AI recommendations conflict with clinical judgment.^80^ Concurrent with these adoption challenges are unresolved medicolegal questions surrounding AI-assisted clinical decisions, including liability attribution in cases of algorithmic error, requirements for informed patient consent regarding AI involvement in care decisions, and the evidentiary standards for AI-generated recommendations in legal proceedings.^81^ Data privacy and security concerns further complicate implementation, as AI systems typically require access to extensive patient data that must be protected according to evolving regulatory frameworks while enabling the cross-institutional data sharing necessary for algorithm validation and improvement.^82^ The economic dimension of AI implementation presents additional complexity, as cost-benefit analyses must account for substantial upfront investments in infrastructure, training, and validation alongside uncertain long-term returns that vary significantly across different healthcare delivery models and patient populations.^83^, ^84^

## Future research directions

The integration of artificial intelligence into perioperative care represents a paradigmatic shift in medical practice, yet its full potential remains to be realized. As we stand at this critical juncture, three interconnected pillars emerge as fundamental to advancing this field: the development of comprehensive multimodal benchmark datasets as essential infrastructure, the evolution of human-AI collaborative decision-making paradigms as the operational framework, and the establishment of robust ethical and regulatory frameworks as the societal foundation. These pillars form a synergistic ecosystem that will determine the trajectory of AI implementation in perioperative care. Their interdependence underscores the necessity of concurrent advancement across all three domains to achieve meaningful clinical impact while ensuring responsible innovation. The following sections explore these critical directions in detail, examining both their individual significance and their collective role in shaping the future of AI-enhanced perioperative care.

### Construction of multimodal benchmark datasets

The establishment of standardized multimodal benchmark datasets represents a critical infrastructure imperative for advancing perioperative AI applications. Recent developments in comprehensive perioperative datasets, such as INSPIRE^85^ and the University of Queensland Vital Signs Dataset,^61^ have demonstrated the feasibility and value of capturing heterogeneous data streams, including physiological signals, clinical records, and medication data. These pioneering efforts have highlighted the essential role of standardized data collection and preprocessing protocols in ensuring data quality and research reproducibility. Moreover, successful implementations of multimodal integration, as evidenced in nociception monitoring studies^86^ and sepsis prediction challenges,^87^ underscore the potential of well-curated datasets to facilitate algorithmic evaluation standardization and cross-center validation. This momentum towards open science, exemplified by the systematic review of perioperative open datasets,^88^ is catalyzing unprecedented collaboration between academia and industry, while simultaneously establishing robust frameworks for model comparison and validation across diverse clinical settings.

The critical importance of external validation across diverse clinical populations cannot be overstated in the development of generalizable perioperative AI systems. However, current validation studies often suffer from limited demographic representation, as most algorithms are trained and tested on data from high-resource academic medical centers in developed countries, potentially limiting their applicability to broader patient populations.^89^ This demographic homogeneity raises significant concerns about algorithmic performance across different ethnic groups, socioeconomic backgrounds, and healthcare settings with varying resource constraints.^90^ Recent analyses have revealed substantial performance degradation when AI models developed in one institution are deployed in different clinical environments, with accuracy drops of 10–25 % documented in cross-institutional validations.^91^ To address these limitations, future research must prioritize multicenter prospective validation studies that deliberately include diverse patient cohorts across geographic regions, healthcare systems, and demographic characteristics. The integration of real-world data (RWD) from routine clinical practice, rather than curated research datasets, presents a promising avenue for enhancing external validation robustness.^92^ Federated learning approaches, which enable model training across multiple institutions without centralizing patient data, offer a technically feasible solution for large-scale validation while preserving data privacy.^93^ Additionally, the establishment of international validation consortiums, similar to successful models in genomics research, could facilitate systematic evaluation of perioperative AI tools across diverse global populations, ensuring that advances in AI-enhanced perioperative care benefit all patient populations equitably rather than exacerbating existing healthcare disparities.^94^

### Human-AI collaborative decision-making paradigm

The evolution of human-AI collaborative decision-making in perioperative care represents a paradigmatic shift from traditional binary approaches of either complete automation or purely human judgment. Contemporary evidence from systematic analyses of AI-assisted interventions^95^ demonstrates the clinical utility of this collaborative paradigm, particularly in critical scenarios such as hypotension prediction and management. The complementary synergy between AI's computational capabilities and clinicians' experiential knowledge has emerged as a fundamental principle,^96^, ^97^ as evidenced by AI's capacity to process vast multimodal perioperative datasets for rapid pattern recognition while anesthesiologists provide critical contextual interpretation and clinical reasoning.^74^ This symbiotic relationship manifests particularly in anesthesia depth monitoring and event prediction, where AI algorithms excel at continuous high-dimensional data analysis while clinicians leverage their expertise to validate predictions and make nuanced therapeutic adjustments.^77^ This symbiotic relationship enables dynamic adaptation of interaction levels based on situational complexity, while maintaining decision transparency and clear accountability structures.^98^, ^99^ Notably, recent investigations into machine learning applications in anesthesiology^100^ have illuminated the potential for AI to augment, rather than replace, clinical decision-making by providing real-time data analysis and predictive insights, thereby establishing a framework where human expertise and artificial intelligence converge to optimize perioperative care delivery.

### Ethical and regulatory framework

Implementing AI technologies within perioperative care necessitates rigorous ethical and regulatory frameworks to ensure patient safety and system integrity. Privacy protection remains paramount as AI systems process sensitive patient data, requiring adherence to established regulations while implementing enhanced security measures such as federated learning and differential privacy techniques.^101^, ^102^ Algorithmic fairness presents another critical challenge, as AI models trained on homogeneous datasets may perpetuate or amplify existing healthcare disparities across patient subpopulations, demanding diversity in training cohorts and continuous bias monitoring.^77^, ^101^ The attribution of responsibility in AI-assisted clinical incidents continues to evolve, with current paradigms emphasizing the anesthesiologist's supervisory role and ultimate clinical accountability despite AI involvement.^64^, ^103^ Regulatory standards for AI-based medical devices are rapidly developing globally, with many jurisdictions adapting existing Software as Medical Device (SaMD) frameworks to address the unique characteristics of self-learning AI systems, including their adaptive algorithms and continuous performance evolution based on real-world data exposure.^77^, ^102^ As these technologies advance, a harmonized international regulatory approach similar to emerging voluntary codes of conduct may facilitate innovation while maintaining stringent safety standards.^102^

## Conclusions

Despite the comprehensive scope of this review, several limitations should be acknowledged. First, the rapid evolution of AI technologies in perioperative care means that some recent developments may not be fully captured within our review timeframe, potentially limiting the currency of certain technical discussions. Second, while we systematically analyzed the literature, the heterogeneity in study designs, patient populations, and evaluation metrics across different institutions makes direct comparisons challenging and may affect the generalizability of our findings. Finally, the limited availability of large-scale, multicenter clinical implementation data restricts our ability to provide definitive conclusions about the real-world effectiveness and safety of these AI systems in diverse perioperative settings.

This review documents the paradigm shift from static decision frameworks to dynamic, adaptive systems in perioperative care. The integration of multimodal data fusion with closed-loop optimization establishes a new anesthesiology paradigm characterized by real-time data integration and adaptive decision optimization. The literature indicates three critical implementation dimensions: computational optimization under real-time constraints, performance robustness across clinical scenarios, and interpretable decision frameworks. This transition from static to dynamic perioperative care represents a fundamental reconceptualization of clinical decision architecture that leverages both computational capabilities and clinical expertise, promising unprecedented precision while establishing new standards for patient safety and clinical efficiency.

## CRediT authorship contribution statement

**Zhewei Ye:** Writing – review & editing, Funding acquisition, Resources, Supervision. **Xiangxin Lv:** Data curation, Formal analysis, Investigation. **Mingdi Xue:** Writing – original draft, Conceptualization, Methodology, Writing – review & editing. **Rui Luo:** Investigation, Writing – review & editing. **Weihang Gao:** Data curation, Formal analysis. **Xiaoliang Chen:** Investigation, Resources, Validation. **Zineng Yan:** Formal analysis, Investigation, Writing – review & editing. **Honglin Wang:** Data curation, Investigation, Validation. **Jiaming Yang:** Data curation, Formal analysis, Investigation, Writing – review & editing. All authors have read and agreed to the published version of the manuscript.

## Consent for publication

Not applicable.

## Ethical statement

Not applicable.

## Funding

This research received no external funding.

## Declaration of competing interest

The authors declare that they have no known competing financial interests or personal relationships that could have appeared to influence the work reported in this paper.

## Data Availability

All data cited in this review are publicly available through the original publications listed in the references. Source data for figures are provided with this paper.
